# 

**Published:** 1886-11

**Authors:** 


					﻿Entered at the Post Office at Austin. Teras, as Second-Class Manil Matter.
Vol. II
NOVEMBER. 1886.
No. 5.
DANIEL'S
MEDECAL
JOURNAL
F. E DANIEL, M. D., Editor and Publisher. AUSTIN TEXAS.
Northern Office, 1217 Filbert Street, Philadelphia.
Eugene Von Boeckmann. Steam Book and Job Printer. Aurtin, Texas.
				

